# Supplementary material for: Enhancing Resistance to Cercospora Leaf Spot in Mung Bean (Vigna radiata L.) through Bradyrhizobium sp. DOA9 Priming: Molecular Insights and Bio-Priming Potential
Source: Plants (Basel). 2024 Sep 5;13(17):2495. doi: 10.3390/plants13172495 (PMC11396852; doi:10.3390/plants13172495)
Supplement: Supplementary file 1 [file plants-13-02495-s001.zip › Susplemantaly data Table-Enhancing resistance to Cercospora leaf spot in mung bean (Vigna radiata L.) through Bradyrhizobium sp. DOA9 priming Molecular insights and biocontrol potential.pdf]

Table S1. The relative gene expression of *V. radiata* CN72 leaf at 1 dpi.

Genes	Relative genes expression at 1 dpi							
	NI	LP	SP	DP	NI+CC	LP+CC	SP+CC	DP+CC
NDR1	1.02±0.15	3.65±1.70	2.67±0.94	4.14±1.94	6.89±0.91	4.60±0.33	↑24.12±3.61*	↓3.61±0.26*
EDS1	1.06±0.07	0.80±0.24	0.81±0.04	1.21±0.34	0.98±0.06	↑2.33±0.17*	0.90±0.05	1.26±0.15
Pti5	1.00±0.03	1.47±0.37	1.39±0.11	1.53±0.17	0.66±.09	↓0.20±0.02*	↓0.35±0.05*	0.74±0.19
Pti6	1.01±0.12	1.12±0.36	↓0.38±0.07*	↓0.38±0.06*	0.25±0.01	↑0.64±0.04*	0.29±0.02	0.39±0.12
PR-1	1.19±0.40	0.61±0.30	0.20±0.03	0.64±0.18	0.47±0.11	0.32±0.02	0.47±0.19	↑13.19±3.07*
PR-2	1.25±0.46	↑4.08±0.43*	↑8.04±2.07*	↑13.04±4.66*	12.97±0.45	↓1.50±0.46*	20.10±5.55	↓3.14±1.29*
PR-5	1.01±0.12	↓0.00±0.00*	0.89±0.37	1.44±0.32	0.04±0.01	0.00±0.00	↑2.10±0.52*	0.00±0.00
PR-3	1.02±0.13	0.90±0.15	1.37±0.26	5.66±1.92	2.76±0.54	1.50±0.46	6.20±2.22	11.01±4.19
PR-4	1.07±0.25	↓0.04±0.00*	0.65±0.06	0.65±0.32	19.24±2.54	25.48±2.11	↓3.67±0.12*	↓2.99±0.78*
CHS	1.04±0.18	1.36±0.69	1.08±0.30	1.41±0.74	13.31±2.37	↓2.65±0.86*	↓4.70±1.35*	17.60±7.43
Pti1	1.01±0.13	0.52±0.10	↑2.00±0.27*	0.43±0.12	7.50±1.62	7.86±1.22	↑17.88±3.33*	12.49±1.59
Prx	1.07±0.26	1.26±0.64	1.22±0.13	0.59±0.21	19.43±2.44	17.42±3.66	24.07±2.82	22.31±6.53
HR	0.98±0.15	0.86±0.21	1.08±0.20	0.98±0.25	6.04±1.58	5.09±2.06	1.89±0.42	2.30±1.20

Data represent mean ± SEM; N = 3. Statistical analysis for each group was performed by comparing with non-inoculated controls (NI) for non-pathogen infection or NI+CC for pathogen infection. Symbols indicate statistical significance (t-test, $p < 0.05$).

Table S2. The relative gene expression of *V. radiata* CN72 leaf at 2 dpi.

Genes	Relative genes expression at 2 dpi							
	NI	LP	SP	DP	NI+CC	LP+CC	SP+CC	DP+CC
NDR1	1.08±0.31	↑17.69±3.93*	2.21±0.32	1.32±0.31	132.78±16.7	↓61.96±13.43*	↓27.34±1.24*	↓42.50±7.20*
EDS1	1.03±0.17	↑10.38±0.90*	↑2.31±0.06*	1.72±0.62	6.99±0.40	7.22±1.06	7.47±0.61	↑14.22±0.53*
Pti5	1.01±0.08	↑7.40±0.79*	1.11±0.09	↑5.53±1.07*	5.49±0.80	↑10.52±1.30*	8.75±1.33	8.60±1.27
Pti6	1.01±0.09	↑4.99±1.34*	1.53±0.27	5.29±1.63	5.02±0.48	↑11.23±0.55*	↑12.38±1.79*	8.75±1.55
PR-1	0.00±0.00	0.00±0.00	0.00±0.00	0.00±0.00	0.00±0.00	0.00±0.00	0.00±0.00	0.00±0.00
PR-2	1.07±0.28	0.56±0.21	0.61±0.19	1.29±0.10	13.55±2.65	13.77±2.89	8.81±2.39	9.58±1.75
PR-5	1.07±0.28	3.59±1.37	0.88±0.19	1.15±0.18	0.20±0.09	↑2.01±0.32*	1.22±0.48	2.63±1.00
PR-3	1.10±0.30	2.69±1.07	0.48±0.09	10.54±5.39	37.84±3.73	↓15.30±1.96*	↓7.90±0.71*	↓14.36±2.83*
PR-4	1.03±0.8	6.97±4.94	2.14±1.02	4.45±1.65	142.12±28.16	83.66±13.50	185.45±29.16	148.16±21.45
CHS	1.07±0.25	↑3.50±0.08*	1.52±0.20	↑4.46±1.14*	61.40±8.35	58.49±10.74	62.52±24.82	52.15±14.87
Pti1	1.02±0.14	↑9.76±1.48*	↑6.12±1.22*	↑13.77±0.87*	44.55±1.33	↓25.77±5.35*	↓18.20±2.03*	↓19.80±3.18*
Prx	1.30±0.65	3.17±0.29	1.03±0.16	1.91±0.36	29.21±2.62	↑85.91±10.49*	↑82.41±11.44*	↑59.27±4.82*
HR	1.07±0.25	↑3.63±0.71*	1.56±0.25	1.81±0.45	36.60±4.26	↓12.54±2.74*	↓11.93±4.35*	↓15.82±4.68*

Data represent mean ± SEM; N = 3. Statistical analysis for each group was performed by comparing with non-inoculated controls (NI) for non-pathogen infection or NI+CC for pathogen infection. Symbols indicate statistical significance (t-test, $p < 0.05$).
