# Supplementary figures and images for: Enhancing Resistance to Cercospora Leaf Spot in Mung Bean (Vigna radiata L.) through Bradyrhizobium sp. DOA9 Priming: Molecular Insights and Bio-Priming Potential
Source: Plants (Basel). 2024 Sep 5;13(17):2495. doi: 10.3390/plants13172495 (PMC11396852; doi:10.3390/plants13172495)

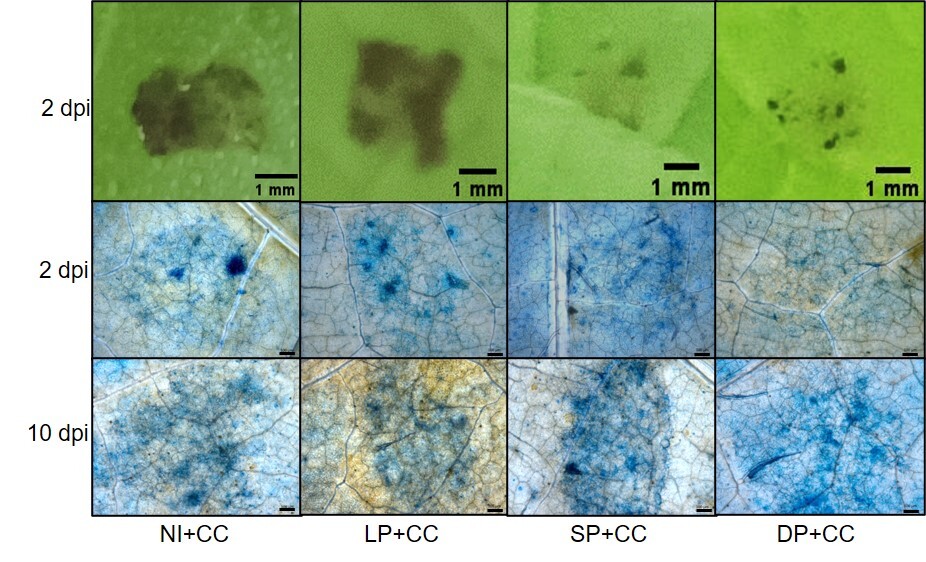

Supplement: Supplementary file 1 [file plants-13-02495-s001.zip › Figure S2..jpg]

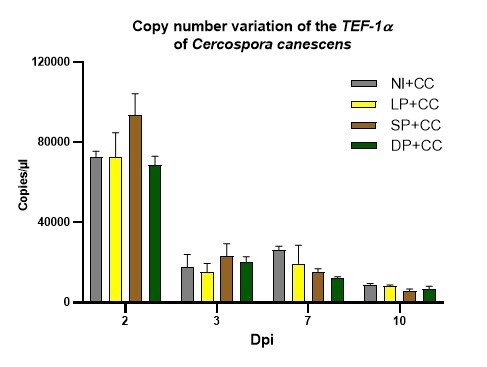

Supplement: Supplementary file 1 [file plants-13-02495-s001.zip › Figure S1..jpg]
